# Assessing arrays of multiple trail cameras to detect North American mammals

**DOI:** 10.1371/journal.pone.0217543

**Published:** 2019-06-17

**Authors:** Bryn E. Evans, Cory E. Mosby, Alessio Mortelliti

**Affiliations:** 1 Department of Wildlife, Fisheries, and Conservation Biology, University of Maine, Orono, Maine, United States of America; 2 Maine Department of Inland Fisheries and Wildlife, Bangor, Maine, United States of America; Cornell University, UNITED STATES

## Abstract

Motion triggered camera traps are an increasingly popular tool for wildlife research and can be used to survey for multiple species simultaneously. As with all survey techniques, it is crucial to conduct camera trapping research following study designs that include adequate spatial and temporal replication, and sufficient probability of detecting species presence. The use and configuration of multiple camera traps within a single survey site are understudied considerations that could have a substantial impact on detection probability. Our objective was to test the role that camera number (one, two or three units), and spacing along a linear transect (100 m or 150 m), have on the probability of detecting a species given it is present. From January to March, 2017 we collected data on six mammal species in Maine, USA: coyote (*Canis latrans*), fisher (*Pekania pennanti*), American marten (*Martes americana*), short-tailed weasel (*Mustela erminea*), snowshoe hare (*Lepus americanus*), and American red squirrel (*Tamiasciurus hudsonicus*). We used multi-scale occupancy modelling to compare pooled detection histories of different configuration of five cameras deployed at the same survey site (n = 32), and how the configuration would influence the probability of detecting a species given it was available at the site. Across all six species, we found substantial increases in probability of detection as the number of cameras increased from one to two (22 to 400 percent increase), regardless of the spacing between cameras. For most species the magnitude of the increase was less substantial when adding a third camera (4 to 85 percent increase), with coyote and snowshoe hare showing a pronounced effect. The influence of survey station features also varied by species. We suggest that using pooled data from two or three cameras at a survey site is a cost effective approach to increase detection success over a single camera.

## Introduction

Motion-triggered camera traps are an increasingly popular tool for wildlife research [1,2]. Early camera trapping studies initially built upon existing capture-mark-recapture analysis-framework [3], but the approach has since expanded to study unmarked individuals ranging from the species to the community level [4,5]. Camera trapping studies have been proposed as a tool for large-scale, long-term monitoring [6], and are an important development for terrestrial wildlife research [7,8]. Data collected by camera trapping can be analyzed in an occupancy modeling framework [9], where consecutive days or weeks of data collection create a detection history of animal visits but do not require the researchers to be present at all occasions. Originally designed for distinct patches of habitat, occupancy modeling has been expanded to include use in continuous habitat, where the activity patterns of animals that bring them into the detection range of a remote sensor correspond to presence in the area [10,11,12]. Occupancy modeling is a flexible, statistically robust approach which accounts for biases in parameter estimates caused by false-absences or temporary unavailability [13]. The approach offers further biological insights by modeling covariates [9] and provides ecologically relevant information to researchers and managers [14].

Study design is a fundamental step in any wildlife research project. Factors to consider include selecting an appropriate scale and study sites that meet the goals of the study, adequate spacing between sites for independence, and the trade-off between effort invested at every survey site (often the number of visits to a site, or duration of camera deployment) and the total number of sites [15,16]. When considering the study objective, there are also trade-offs between addressing specific questions regarding one, or a few, focal species versus collecting broader data at a community level [17,18]. An approach which may maximize efficacy for some species may not be optimal for all, and to implement an effective study design information on the actual impact of these trade-offs is essential.

The specific aspect of study design we investigate below is the use of multiple camera traps, placed at unique microsites but within in a single survey site, to increase the cumulative detection probability of target species. While there has been research to address the trade-off between total number of camera survey sites in a study area and deployment time at each [19], relatively fewer studies have assessed the impact of increasing the number of cameras contributing data to a single survey site. Increasing the number of cameras, and the heterogeneity of the available habitat surveyed by placing them in distinct microsites, should increase detection probability. Although it is not the goal of most research studies itself, having a reasonable chance of successfully detecting species when they are present is essential to avoid biased results [15,16]. This is especially important for species that occur at low density, such as carnivores [20]. Recently, O’Connor and colleagues [21] assessed the benefits of increasing number of cameras in randomized clusters for four unmarked North American mammal species, and did find a dramatic gain in detection probability when more than one camera was used. Similarly, researchers in Australia found an increase in detection for feral cats when increasing the number of cameras in a cluster up to five [22]. These previous studies, however, used grouped or random arrangements. A study design using a consistent arrangement and spacing of cameras at each study site would reduce variation between sites but allow each site to include different small-scale landscape features, and may increase the likelihood of detecting organisms with clustered distributions [23] or activity patterns that are not perfectly uniform across the landscape. This movement behavior is expected for animals seeking optimal foraging opportunities, avoiding predation, and moving throughout a heterogeneous home range [24,25,26,27,28]. Our aim was to help fill this knowledge gap by comparing the detection success (defined as a higher likelihood of observing the species if it is available in the area, or conversely lower chances of a false absence) between camera trap survey methods that differed in the number of cameras deployed at one, two or three separate microsites within each survey site and the spacing between them. This differs from the use of multiple cameras at one single location to assess missed detections [29] or to assist individual identification of animals with unique markings (such as [30]).

Our study used multi-scale occupancy models to compare detection probabilities between survey methods that varied in the number (one, two or three) and spacing (100 m or 150 m for transects of two- and three-cameras) of cameras set in a “T” arrangement at a single survey site. This approach is a subset of occupancy modeling designed to compare efficacy of different data-collection techniques while accounting for non-independence of co-located devices or detection methods [31]. Though prior research has implemented a multi-scale approach using camera trap data, these studies typically compare cameras to other survey techniques (such as track plates, snow tracking or genetic capture-recapture [32,33]). Our objectives were 1) to use a multi-scale approach to determine the most effective arrangement of camera traps in terms of both spacing and number of cameras deployed to increase detection probability and 2) to exploit data from multiple species collected at the same sites to illustrate how the optimal study designs may vary between species. Our primary goal is to investigate, on behalf of future research, how much is gained in increased detection probability at a single survey site by using multiple cameras. By placing cameras in close proximity but at unique microsites to attract or opportunistically detect wild mammals, and by pooling the resulting detecting histories, we predict that the risk of false-absences is decreased and therefore stronger inferences can be drawn.

We analyzed the detection histories of six terrestrial mammal species: coyote (*Canis latrans*), fisher (*Pekania pennanti*), American marten (*Martes americana*), short-tailed weasel (*Mustela erminea*), snowshoe hare (*Lepus americanus*), and American red squirrel (*Tamiasciurus hudsonicus*). These species span across a range of trophic levels and body sizes, which impact movement patterns and space use and therefore the detection probability during camera surveys. The carnivore species represent both the cursorial hunting strategy of coyotes and the more opportunistic, exploratory mustelids [25,26] which could influence the likelihood of detection. Even with a baited site, a broader camera spacing might increase the chances that at least one camera fell along the movement path of a coyote, where a denser spacing might increase the overall attractant and draw mustelids closer as they investigate their surroundings. Similarly snowshoe hare are likely to cover greater distances than red squirrels, and thus a longer spacing might increase detection success if this were a target species. By incorporating site covariates that were likely to play a role in animal occupancy and detection, we began to assess how the transect design may have differing impact on the ability of a given survey to detect a target species if it is present.

## Study area

**Fig 1:**
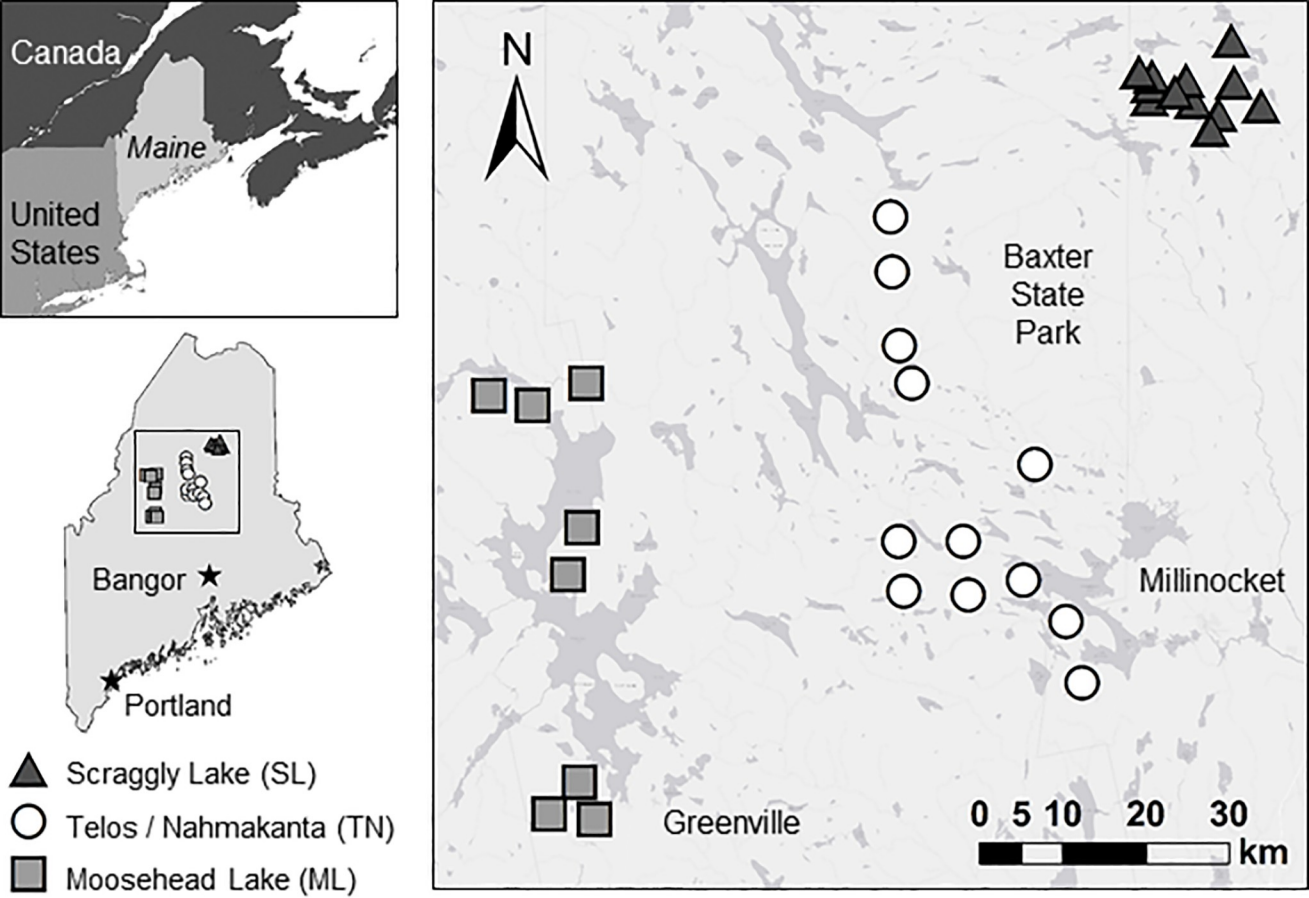
Three study areas surveyed with multiple camera traps in Maine, USA. 12 sites each composed of five camera traps were deployed in the Scraggly Lake (SL) area during January 2017, 12 sites in the Telos Road/Nahmakanta Public Reserve Lands (TN) from February to March 2017, and 8 sites in the Moosehead Lake (ML) area from late February to March 2017.

Our study took place in central and northern Maine at the transition zone of the Eastern Deciduous Forest and the Boreal Forest [34,35] (Fig 1). We surveyed in three study areas: Scraggly Lake (100 km^2^ centered at 46.2455° N, -68.7348° W), Telos Road and the Nahmakanta Public Reserve Lands (430 km^2^, 45.9163° N, -69.1564° W) and northeastern Moosehead Lake (340 km^2^, 45.7073° N, -69.6910° W). These three areas are spaced a minimum of 30 km apart, to capture potential geographic variation in animal distribution patterns over north-central Maine. The region receives precipitation year round (mean annual precipitation 112 cm) and experience mild summers and cold winters (mean summer temperature 18° C, winter -8° C) [36]. All three study areas are heavily forested and are comprised of a mosaic of different age forest stands resulting from recent timber harvest, renewing stands and preserved areas. Coniferous trees are predominant, and include balsam fir (*Abies balsamea*), spruces (*Picea spp*.), and white pine (*Pinus strobus*), while mixed or fully hardwood stands include white and yellow birches (*Betula papyrifera* and *B*. *alleghaniensis*), red and sugar maples (*Acer rubrum* and *A*. *saccharum*), and American beech (*Fagus grandifolia*).

## Methods

### Field methods

**Fig 2:**
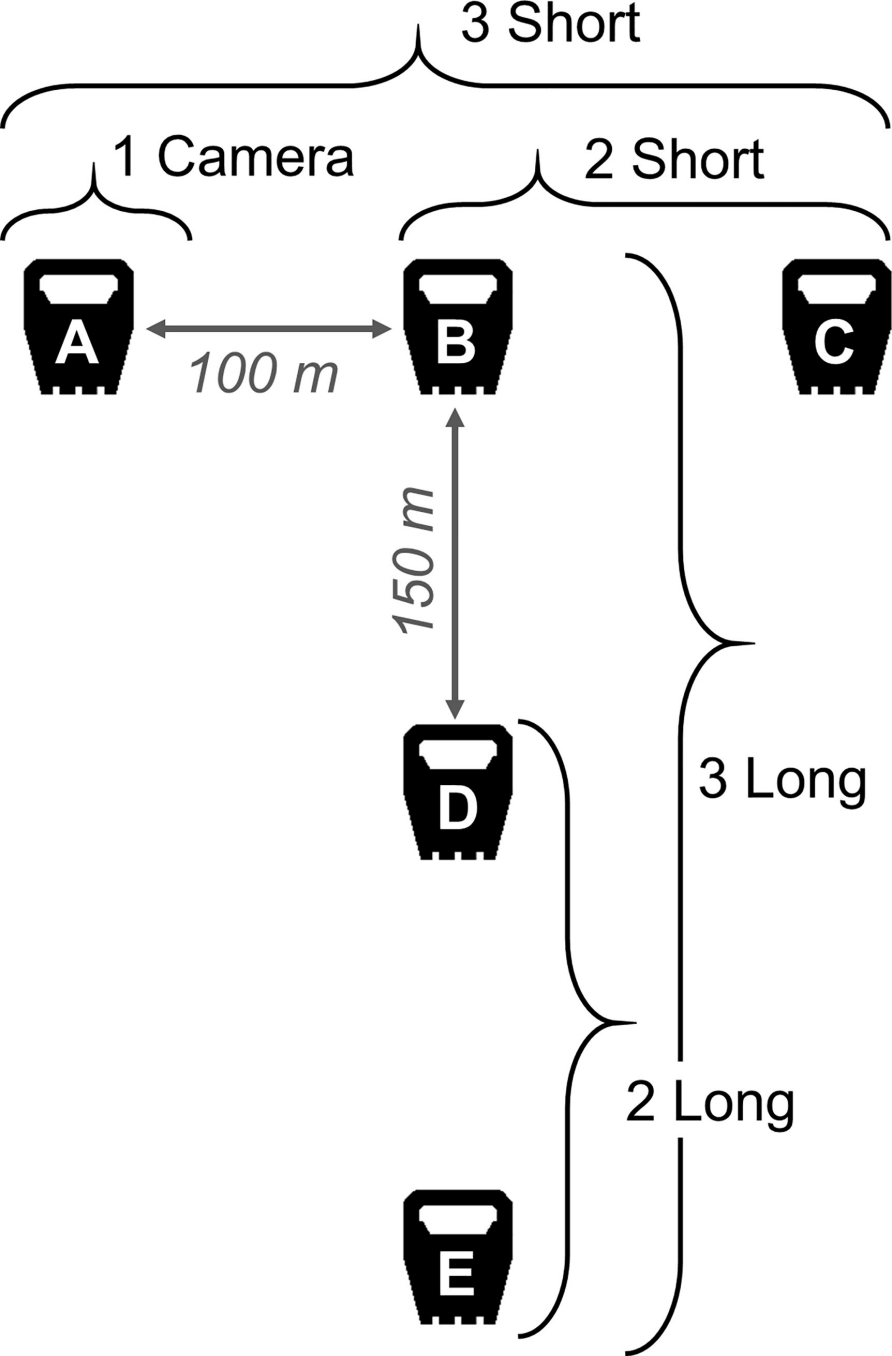
A survey site composed of five camera trap units in a T-configuration to detect terrestrial mammals in Maine, USA. Detection histories from individual cameras were pooled in five different combinations to assess the impact of survey methods that varied by number of cameras (1–3) and spacing (short, 100 m, or long, 150 m between cameras): 1 unit (camera A), 2 short (cameras B and C), 3 short (A, B, and C), 2 long (D and E) 3 long (B, D, and E).

We deployed camera trap survey sites across a range of forest stand ages and management regimes in northern Maine to detect terrestrial mammal species during winter 2017. We used randomized locations, generated prior to conducting field work, to avoid bias in habitat sampled. We traveled as close as possible to these locations by either truck or snowmobile before hiking off of the motorized trail to set cameras. Each site (*sensu* [13]) was sampled with a detection array (*sensu* [31]) which consisted of five cameras arranged in a T-configuration (Fig 2). This configuration allowed us to test how detection probability was impacted by different arrangements of cameras, placed at nearby microsites and thus dependent in terms of habitat characteristics and underlying species occupancy status [30]. We consider our sampling unit to be each site with five camera and spaced these to be independent of one another in terms of overall species use of the surrounding habitat (goal distance of 5 k between nearest sites, mean 5.1, range 1.6–12.7).

Three cameras were spaced in a straight line 100 m apart, and two cameras were spaced 150 m perpendicular from the central camera. Each camera microsite consisted of one Bushnell Trophy Cam Essential 2 passive infrared (PIR) triggered camera (Overland Park, KS, USA) attached to a tree at approximately 1 m above snow level. The site was baited with a portion of beaver (*Castor canadensis*) meat enclosed in a suet cage wired to a tree 3–4 m in front of the trail camera and scent lure designed to attract furbearers (skunk essence and Vaseline based, Kenduskeag, ME, USA). Cameras were programed to take a single image for every PIR trigger event, and to record time-lapse images at 03:00 and 15:00 to capture weather events impacting performance. The microsite and the placement of cameras were chosen to optimize unimpeded access to, and visibility of, the bait. We sought out natural clearings and likely movement corridors to increase the probability of animals visiting a site. We deployed camera trap arrays for two to three weeks from January to March, 2017. We did not rebait the sites, instead using the suet cage to prevent meat bait from being consumed, and revisited to retrieve the cameras and reset in the next study site. The techniques implemented in this non-invasive wildlife research study were presented to the University of Maine Institutional Animal Care and Use Committee and were approved under Protocol A2018-05-06.

### Analytical methods

Detection histories were created for six species of terrestrial mammals at each camera unit. All images recorded in a single day were collapsed into a daily detection result of 0 (not detected) or 1 (detected at that camera). Daily detections were used following similar, relatively short-term camera trapping studies [19, 37]. Although many arbitrary cut-offs have been used in the camera trapping literature [2] a 24-hour period captures natural movement patterns of activity for wildlife species. Non-independence between time units may occur regardless of binning, and our data did not indicate a strong trend at 24 hours. This approach also guaranteed enough revisits over the two- to three-week study duration to avoid models without solutions [15,16], and when we tested for an impact of 24-hours versus 1-week bins, trends were the same for detection results but model fit was poorer in the collapsed dataset (see S1 and S2 Files). Any days in which a camera was not active or was blocked by snow were considered ‘missing visits’ (*sensu* [13]).

We generated pooled detection histories for different combinations of camera numbers and transect lengths, here after referred to as methods, as follows: method 1 = 1 camera, method 2 = 2 cameras short (100 m between cameras), method 3 = 3 cameras short, method 4 = 2 cameras long (150 m between cameras) and method 5 = 3 cameras long (Fig 2). We pooled the individual camera detection histories so that for each method 0 indicates no detections and 1 indicates a detection at any one or more camera in that method. This allowed us to investigate the impact that adding cameras to a survey configuration would have on the probability of detecting a species. One caveat is that our methods using all three cameras for each potential spacing encapsulate the detection histories for the two-camera method, and for the short array, the single camera method. This results in mathematical dependency between our methods, because for methods with three cameras to result in a detection, that event is also captured by at least one other. However, this reflects the application of the techniques, and because the magnitude of difference in detection success going from one or two cameras to three can only be under-estimated we feel our conclusions are conservative and consistent with the underlying detection process.

We fitted multi-scale occupancy models [31] to detection history data for each of the six species considered in this study. The parameters of the multi-scale model are: Ѱ_*i*_, the large-scale probability that a member of the species *i* occupies a home range and uses the landscape surrounding the survey site; ϴ_*it*_, the small-scale probability of a species being in the immediate area of the detection methods on survey occasion *t* given that it occupies the overall area; and p_*it*_, the probability of detection on occasion *t* given that both the overall area is occupied and the species is in the immediate area of the survey devices. In our study, Ѱ is the overall occupancy surrounding all five cameras comprising the survey site, ϴ is the daily probability that a species is within detection range of the cameras, and p is modeled such that each of the different methods of pooling cameras can have a different probability of detecting a species. In the example below, we show the formula for estimating these parameters for two days of a detection history, allowing the method type to influence detection. We have L = 5 methods, over a two-day detection history of H = 01100 00000. This indicates that on day one, detection methods two and three recorded the species, and on day two, no methods recorded an image:

$$\mathrm{Pr}\left(00101\;00000\right)=\Psi \left[\theta_{1}\left(1-p_{1}^{1}\right)(p_{1}^{2})(p_{1}^{3})\left(1-p_{1}^{4}\right)\left(1-p_{1}^{5}\right)\left[\left(1-\theta_{2}\right)+\theta_{2}\prod_{s=1}^{L}\left(1-p_{2}^{s}\right)\right]\right]$$

On day one, we know that the species was available for detection because methods two and three were successful, and can therefore write the other three methods as one minus the probability of detection (known “false-absences”). On day two, no methods detected the species, thus the probability is modeled as the sum of two potential causes: the species was not in the immediate vicinity of the survey sites or all five methods *s* (*sensu* [31]) failed to detect the species. Because at each survey site overall, the probability of large-scale occupancy and small-scale, daily availability is shared, the variation in detection success depends only on each method and any method specific covariates. Models were fitted through Program PRESENCE [38] and were ranked based on the Akaike Information Criteria corrected for small sample sizes (AICc).

We followed a three-stage approach to fit occupancy models. In the initial stage we compared two models that differed only by the inclusion or exclusion of the method type on the detection process. In the second stage we focused on additional covariates affecting detection probability. We included the following covariates (Table 1) which are likely to impact the overall occupancy of a species at a survey site or the detection process within an array: the study area (Area) in which the array was deployed (Scraggly Lake, SL; Telos Road and Nahmakanta Public Reserve Lands, TN; Moosehead Lake, ML) to control for differences due to latitude and longitude and any other unmeasured variables beyond the scope of our camera surveys. The access type (Access) was included as a surrogate for the influence of transportation infrastructures which has been demonstrated to influence detection [39,40,41] and was defined as either road (RD) if the array was located off of a plowed road or snowmobile (SM) if it was accessible only by snowmobile. We also included variables for the distance to the access feature, either road or trail, of the 1–3 camera units within each method. Distance values (DistAve, DistMax, DistMin) were standardized by z-scoring [42]. Following Burnham and Anderson [43] we considered variables included in the top ranked model and any models within 2 ΔAICc (corrected for our small sample size of 32 independent stations) to be important, and tested additive models for these. We retained one top model for the detection process for each species to use in the third and final stages.

**Table 1:** Definitions of terms used in study design and modeling to assess the influence of different numbers and spacing of camera traps on the detection of mammals in Maine, USA.

**Study design**	
Survey site	An arrangement of five baited cameras traps as shown in Fig 2. Each survey site is considered independent of other sites in terms of species occupancy status.
Method	Arrangement of 1, 2, or 3 camera microsites spaced either 100 m (short) or 150 m (long) apart. The five detection methods compared are method 1 = 1 camera, method 2 = 2 cameras short, method 3 = 3 cameras short, method 4 = 2 cameras long and method 5 = 3 cameras long.
Camera	One Bushnell HD motion-triggered camera trap, placed facing bait and lure for two to three weeks.
**Model parameters**	
Psi (Ѱ)	The probability of occupancy at the large scale, here defined as at least one individual of a species using the habitat surrounding a site over the course of the survey and thus having some potential for detection on any survey day (*sensu* [8]). Ѱ_*i*_ is the probability for species *i*.
Theta (ϴ)	The probability that at least one individual of a species is using the area immediately around the entire survey site on a given survey occasion, this is the small scale availability for detection. ϴ_*it*_ is the probability that species *i* is available for detection on survey day *t*.
Detection (p)	The probability of detecting a species given that it both occupies the habitat and is present in the vicinity of the survey site (available at both the large and small scale). This probability can vary with survey method *s* and with species and survey occasion, p_*it*_^*s*^.
**Model covariates**	
Access	The infrastructure type used to reach each survey site: plowed road (RD), or snowmobile trail (SM).
Area	Three different study areas: Scraggly Lake (SL), Telos Road and Nahmakanta Public Reserve Lands (TN), and Moosehead Lake (ML).
Distance	The distance from an access feature to each camera in a method. For methods with more than one camera, DistAve is the mean distance, DistMax is the distance of the farthest camera and DistMin is the distance of the closest camera.
Loss	Timber harvest activity generated from raster data on decreases in tree height from 2000 to 2015 [43]. Loss100m, Loss500m, Loss1k and Loss5k are tree loss at increasing spatial scales to correspond to different species body and home range sizes.

In the third stage we focused on covariates affecting occupancy probability at the large scale of animal use (Ѱ) and the small scale immediately around the site (ϴ). We included the Area categorical variable as above, and the degree of tree loss (Loss) to quantify forest harvest events. This was obtained from the Global Forest Watch project cumulative raster data which indicates 30m pixels that over any one year period from 2000 to 2015 experienced a stand-replacing loss of tree canopy cover [44]. Because wildlife species span a wide range of body sizes, and therefore home range sizes, we assessed tree loss at multiple spatial scales by using four circular buffer sizes: 100 m, 500 m, 1 km and 5 km. We used ArcGIS 10.4.1 (ESRI 2016) to generate buffers around each camera site and then merged the individual camera buffers into polygons to provide information about landscape features at each scale. Pixels categorized as open water (www.maine.gov/megis) were excluded from analyses, and the proportion of remaining pixels which experienced tree loss within each buffer size was standardized using z-scoring. Following Fahrig [45] and Sozio and Mortelliti [46] we compared the relative fit of models with different buffer size and retained only the highest ranking buffer size in the final stage. Lastly we modeled each variable for Ѱ only, for Ѱ and ϴ as a single effect, and for Ѱ and ϴ as separate effects.

We checked for spatial autocorrelation between survey sites in the residuals of simple, single season occupancy models in Program R, using package ‘unmarked’ [47]. We used the most parameterized model for detection data at the full array to create spline correlograms in package *ncf* [48] and found no evidence of spatial dependence for any species. To the best of our knowledge there is no established method to assess model fit within the residual structure of multi-scale analyses, thus we estimated c-hat values for the most parameterized single season model [22,31] and did not observe over-dispersion.

## Results

We deployed 32 arrays of five cameras each for two to three weeks (mean 17.2 days, range 16–23) from 12 January to 19 March, 2017. Three cameras failed to record images, and of the 157 cameras included in analyses three had trap nights treated as missing visits due to heavy snowfall or battery failure. We recorded 2,748 total trap-nights of data and captured 8,826 PIR-triggered images. American marten were the most frequently detected species (at n = 71 cameras, and n = 172 trap nights with detections), followed by fisher (n = 47 cameras, n = 92 trap nights) snowshoe hare (n = 40 cameras, n = 134 trap nights), short-tailed weasel (n = 36 cameras, n = 108 trap nights), coyote (n = 28 cameras, n = 34 trap nights) and red squirrel (n = 22 cameras, n = 49 trap nights).

In the first stage of multi-scale occupancy modeling, the inclusion of the method type in the detection process always performed better than the null model (ΔAICc > 8 for all species, see supplemental information S2 File), confirming that the number and spacing of cameras had an important effect on the likelihood of detecting all species. In the second and third stages, the important variables included in all models within ΔAICc > 2 of the top model for detection and occupancy parameters differed between species (Table 2).

**Table 2:** Top-ranked occupancy models for six species surveyed with camera trap transects in Maine, USA, 2017.

Species	Top Model(s)	ΔAICc	AICw
*Canis latrans*	Ѱ(Loss1k), ϴ(.), p(Method+Access)	0	0.34
	Ѱ(.), ϴ(.), p(Method+Access)	0.02	0.33
*Pekania pennanti*	Ѱ(Loss5k),ϴ(Loss5k), p(Method+Access+DistAve)	0	0.60
	Ѱ = ϴ(Area), p(Method+Access+DistAve)	1.58	0.27
*Martes americana*	Ѱ(.),ϴ(.), p(Method+Area+DistMin)	0	0.59
*Mustela erminea*	Ѱ(Loss100m),ϴ(Loss100m), p(Method+Area)	0	0.99
*Lepus americanus*	Ѱ(Loss500m),ϴ(.), p(Method+Area+Access)	0	0.38
	Ѱ(.),ϴ(.), p(Method+Area+Access)	0.43	0.30
*Tamiasciurus hudsonicus*	Ѱ(Area), ϴ(Area), p(Method+Area)	0	0.96

The models shown are the top ranked model using Akaike’s information criterion corrected for small samples and any models within ΔAICc < 2. AICw is the model weight. Ѱ is the probability of occupancy across all stations, ϴ is the daily probability of availability for detection, and p is the probability of detection given availability.

**Fig 3:**
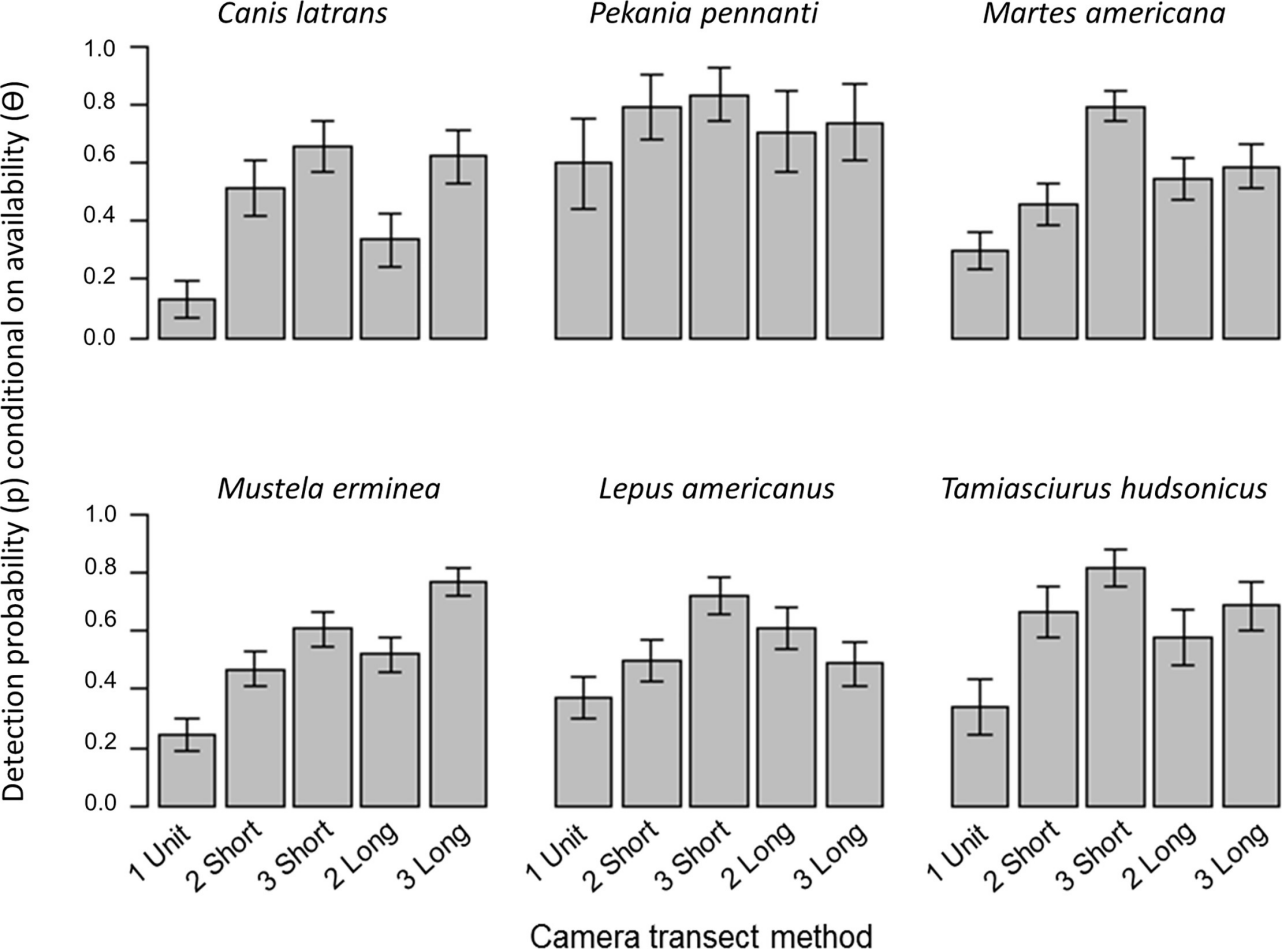
Model-averaged estimated detection probabilities and standard errors for camera trap surveys conducted in north and central Maine, USA, during winter 2017. The detection histories for six species were analyzed for five different potential survey methods of 1–3 camera units spaced short (100 m) or long (150 m). When derived from models including the study area parameter, results are shown for the Telos Road/Nahmakanta Public Reserve Lands, and when models included access type, results are displayed for plowed road access.

For all six species, increasing the number of cameras in a method had a strong positive effect on the detection probability (Fig 3, Table 3). The magnitude of the effect was greatest when increasing from one single camera to two cameras (ranging from 40% increase in detection probability for fisher to 400% for coyote). An increase in detection success was still evident when increasing from two cameras to three (7% to 85%, again for fisher and coyote). The impact of the transect length varied by species, with American marten and snowshoe hare detected slightly more readily at shorter spacing (26% and 32%) and short-tailed weasel showing improved detection success with longer spacing (26%). For coyote, fisher, snowshoe hare and red squirrel, the difference between short and long transects was negligible. We then tested that the differences we observed resulted from the different methods, and not from the arbitrary selection of which two cameras out of the three available were included in the two camera methods. We repeated our analyses using the alternative possible pairs of cameras for two short (units A and B in Fig 2) and for two long (units B and D). We observed very similar trends for all six species in terms of the role that camera number has on detection success. Only the trend for marten and the role of camera spacing varied, as shown in supplemental information Tables A and B in S3 File.

**Table 3:** Parameter estimates for six mammal species in Maine: $\hat{\Psi }$ is the probability of occupancy, $\hat{ϴ}$ is the probability of availability for detection on any survey day, and $\hat{p}$ is the probability of detection with a specific method conditional on availability.

Species	$\hat{\Psi }(\mathrm{se})$	$\hat{ϴ}(\mathrm{se})$	$\hat{p^{s}}(\mathrm{se})$	$\hat{ϴ}∙\hat{p^{s}}$
*Canis latrans*	0.67(0.17)	0.07(0.02)	p^1^: 0.13(0.07)	0.009
			p^2^: 0.51(0.09)	0.036
			p^3^: 0.65(0.08)	0.046
			p^4^: 0.34(0.09)	0.024
			p^5^: 0.62(0.09)	0.043
*Pekania pennanti*	0.73(0.07)	0.12(0.08)	p^1^: 0.60(0.16)	0.072
			p^2^: 0.79(0.11)	0.095
			p^3^: 0.83(0.09)	0.100
			p^4^: 0.71(0.14)	0.085
			p^5^: 0.73(0.13)	0.089
*Martes americana*	0.76(0.08)	0.24(0.02)	p^1^: 0.29(0.07)	0.070
			p^2^: 0.47(0.06)	0.113
			p^3^: 0.79(0.06)	0.190
			p^4^: 0.55(0.07)	0.132
			p^5^: 0.58(0.07)	0.140
*Mustela erminea*	0.51(0.12)	0.33(0.04)	p^1^: 0.24(0.05)	0.079
			p^2^:0.47(0.06)	0.155
			p^3^: 0.60(0.06)	0.198
			p^4^: 0.52(0.06)	0.172
			p^5^: 0.77(0.05)	0.254
*Lepus americanus*	0.50(0.12)	0.36(0.03)	p^1^: 0.37(0.07)	0.133
			p^2^: 0.49(0.08)	0.176
			p^3^: 0.72(0.06)	0.259
			p^4^: 0.49(0.07)	0.220
			p^5^: 0.61(0.07)	0.176
*Tamiasciurus hudsonicus*	0.53(0.31)	0.19(0.10)	p^1^: 0.34(0.10)	0.065
			p^2^: 0.66(0.09)	0.125
			p^3^: 0.81(0.06)	0.154
			p^4^: 0.57(0.09)	0.108
			p^5^: 0.69(0.09)	0.131

Each of five methods of pooling detection histories by different configurations of cameras are compared: p^1^ is a single camera, p^2^ is two cameras spaced 100 m apart, p^3^ is two cameras spaced 150 m, p^4^ is three cameras spaced 100 m and p^5^ is three cameras spaced 150 m. $\hat{\Psi }$ is the estimated probability of occupancy, $\hat{ϴ}$ is the estimated daily probability of availability for detection, $\hat{p^{s}}$ is the probability of detection given availability for each of five camera configuration methods *s*, and se is the standard error. The multiplied value of $\hat{ϴ}$ and $\hat{p^{s}}$ represents the daily detection probability of a single-scale occupancy model with only the data from cameras pooled into that method. When detection was modeled by study area, Telos Road/Nahmakanta Public Reserve Lands estimates are shown; when modeled by access type, plowed road estimates are shown.

Of the parameters influencing the detection process, study area was most frequently included in top ranked models (Table 4). The next most frequently included variable was the access type dominating the camera array. For fisher and snowshoe hare, arrays set near plowed roads showed higher probabilities of detection, while for coyote arrays set near snowmobile trails showed higher detection. The distance to road or trail was important for marten and for fisher, with both species showing an increase in detection probability as transects were placed farther from roads and trails.

**Table 4:** Influence of camera site features on detection probabilities for six mammal species in Maine, USA.

Variable	Species	Beta Estimate
Access type (road)	*Canis latrans*	-0.262
	*Pekania pennanti*	+0.169
	*Lepus americanus*	+0.177
Minimum distance to road or trail*	*Martes americana*	+0.015
Average distance to road or trail*	*Pekania pennanti*	+0.008
Study area	*Martes americana*	SL: *Intercept* TN: -0.037 ML: +0.143
	*Mustela erminea*	SL: Intercept TN: -0.005 ML: -0.529^§^
	*Lepus americanus*	SL: *Intercept* TN: +0.057 ML: -0.192
	*Tamiasciurus hudsonicus*	SL: *Intercept* TN: +0.297 ML: +0.292

Surveys occurred in winter, thus Access type was defined as either plowed road or snowmobile trail. Distance to road varied for different camera arrays, with minimum distances as close as 10 m and median distance up to 320 m. The three study areas were Scraggly Lake (SL), the Telos Road/Nahmakanta Public Reserve Lands (TN), and Moosehead Lake (ML).

*Betas indicate the impact of an additional 10 m in distance from the access point

^§^*Mustela erminea* (short-tailed weasel) was only detected once in the Moosehead Lake study area.

## Discussion

Our findings demonstrate that increasing the number of camera traps deployed within a survey site had a positive effect on the probability of detection for six species of North American mammals, while the distance between cameras was less important. Although the objectives of camera trapping studies can vary widely, if detection probability at each site is low, estimates of occupancy or other parameters of interest are likely to be biased [13,16]. We found that the magnitude of the increase in probability of detection given availability varied, but across all species it was greatest going from a single camera to two cameras at a site. The increase from two cameras to three was most substantial for coyote, the species with the lowest probability of availability for detection during any one survey occasion.

### Camera trap number and spacing

As the price for trail cameras decreases, it is more economically feasible to place more than one unit at distinct micro-sites within a single survey site, without sacrificing the total number of independent sites (though see [49]). Because it is often time and labor intensive to access spatially independent survey sites, we believe it is prudent to follow a study design incorporating multiple cameras placed systematically to improve the effectiveness of each site and to safe-guard against wasted effort in the event of camera failures. While increasing the number of camera units at a site has been demonstrated to increase detection probability [21,22,29], the magnitude of the increase is likely to vary between regions and target species. We found that the increase in detectability was higher going from a single camera to two cameras than when increasing cameras from two to three and, in particular, it was beneficial for species with lower detection probabilities, such as coyote. Although many other components of study design will influence detection success, such as duration of camera deployments and the use and type of baits and lure, the addition of cameras can be considered alongside these. Increasing the number of camera units at each site may help also reduce the need to place cameras for long periods of time (see [19]) which will both increase the overall number of sites that can be sampled within a season and avoid violations of occupancy model closure assumptions [9,10, 50]. The above trade-offs in study design will also depend on the species or group of species targeted and must be considered within the context of overall study objective [17,18]. Increasing the number of camera units is a valuable tool and can provide large gains with low added cost when including the time and effort expended to reach each survey site, and should be considered during the study design process. In particular, our study demonstrates that generally the greatest gain in detection probability can be obtained by working with two cameras, spaced either 100 m or 150 m, as compared to using a single camera.

We concede that the dense arrangement of cameras we deployed may have resulted in a saturation of lure that could have inflated the detection probabilities we observed. The attractant could have increased the daily availability (ϴ) of species with a home range overlapping the survey site, and this would then influence the overall detection probability of our methods. However, we do not expect this to bias the results between different configuration methods because all cameras faced bait and lure. Furthermore, we emphasize that the multi-scale modeling approach accounts for non-independence of different methods being compared [31]. Indeed the dependence between scales is exploited to allow for comparisons between methods at each array thus attributing variation in the detection process to the different number and spacing of camera units [31].

We have expanded upon other recent studies showing the impact of increasing the number of cameras by explicitly investigating how consistent arrangements affect detection success. We observed the highest detection probabilities for American marten and short-tailed weasel in survey methods with 150 m spacing, and the highest detection probability for coyote with 100 m spacing. This may reflect that although weasel and marten are smaller bodied than coyotes, they differ in their hunting strategy and tend to investigate many features of the landscape they move through, rather than following a cursorial route [24,25]. Fisher, red squirrel and snowshoe hare showed almost no difference based on transect spacing. Although our results suggest that transect length has the potential to influence detection and warrants further consideration, the influence of the transect spacing, either 100 m or 150 m, was not as strong as increasing the number of cameras. Furthermore, the difference between 100 m and 150 m, which was the most feasible spacing given the constraints of our study, is relatively small. Although this ensures dependence among the camera sites in terms of animal availability for small- to medium-bodied species, future work investigating greater differences in the lengths between camera units would be valuable. The ideal spacing of camera units is likely to vary in different regions and between species of interest, and one trade-off to consider when designing surveys will be maximizing detection success for one single species versus obtaining adequate, unbiased data on multiple target species. In the context of our work that may mean selecting a transect spacing that raises detection probability for the species with the lower inherent probability, though this same arrangement may not maximize all target species.

### Landscape and camera site features

We found that the site characteristics which we modeled (overall study area, access type, and distance to access feature) influenced the detection process for species differently. For coyote, American marten, and fisher, the access type nearest to the camera transect ranked highly as an explanatory variable for the detection process. Sites off of plowed roads had higher likelihoods of detection for marten and fisher while sites off snowmobile trails had higher likelihoods for detecting coyote. Although not the focus of our study, recreational activity have been shown to influence carnivore behavior [39,51], and this may reflect how different carnivore species perceive and avoid human threats. This is an important consideration when designing camera surveys especially when ease of access to deploy sites could bias results. One further consideration is that season could impact ease of movement for both researchers and wildlife species. Winter has been preferred for some survey methods (e.g. snow track surveys [52], camera traps and genetic sampling [53]) but results could vary by season. For both American marten and fisher, there was also a positive relationship between detection success and increasing distance from the access feature, though even sites close to roads and trails did detect these species. Our individual camera microsites ranged from 10 m to 320 m away from a road or snowmobile trail (mean 133 m). Fisher especially had higher probability of detection for methods located on average farther from the access route, suggesting that researchers should avoid only placing sites close to roadways despite the logistic ease. Similar findings were recently shown in European carnivores [54], suggesting that some, though not all, species avoid close proximity to roadways and that placing survey sites at a range of distances will increase the efficacy of results when working in a multi-species system.

The study areas in which the camera stations were placed spanned three regions in north-central Maine, and this was the most frequently included variable. For most species the Telos Road/Nahmakanta Public Reserve Lands region showed the highest detection probability. American marten, which is a species of special interest in Maine due to possible impacts from land use change [55], was the only species which showed a lower detection probability in this study area. While a detailed comparison between the three study areas is not the goal of this paper, we note that for this species increasing the survey unit from one to two cameras increased detection probabilities to 45–60% across all three of our study areas. However, this does highlight that the detection process will vary by species, and may be important particularly in regions with small populations. Therefore we recommend pilot studies to determine a survey design that will be able to acquire robust data for any high priority target species and avoid ambiguous findings stemming from inadequate study methods (see [56]).

### Camera trapping survey design

As the importance of camera trap data continues to grow, optimizing the efficacy of survey design is in the best interest of both wildlife research and applied management, and increases the potential for collaboration [57]. For all camera surveys, regardless of overall objective, increasing the probability of detection for species of interest when it is indeed present (which can also be thought of as decreasing false-absences) will improve data quality and enable better interpretation of underlying biological processes. For monitoring efforts or research on the underlying causes of species distribution at large scales, increasing the detection probability at each independent survey location by pooling the data from multiple cameras in close proximity, but at unique microsites, will increase the reliability of occupancy estimates [9]. To explicitly clarify, although we have analyzed our data using a multi-scale occupancy model to understand how different configurations of camera can impact detection, we would encourage researchers to use the pooled observation histories of all co-located devices to maximize detection probability at each site as a whole. However it is important to note that recent work [10,11,12] indicate that for mobile organisms such as terrestrial carnivores, “occupancy” of an area may be better described as the probability that an animal’s movement pattern will bring it in front of the relatively small field of few of point sampling devices, such as trail cameras. Even for different research objectives, such as spatial capture-recapture which will require dependence at survey sites themselves, increasing detection success with multiple sites may be worth considering [58,59].

Our findings may be valuable to researchers in North America where these species occur, as well as in similar ecosystems in Europe. For example, the European pine marten (*Martes martes*) and beech marten (*Martes foina*) are sympatric species that may experience competition and exclusion similar to the American marten and fisher in parts of their range [60,61]. Camera trapping research for these species will benefit from using study designs that ensure high quality data on multiple species of interest [62]. Further research on the role that camera number and transect length can have in other ecosystems will be useful, and can be implemented in a pilot season prior to broader scale survey efforts. Particularly if species have relatively high detection probabilities, such pilot seasons could avoid unnecessary increases in survey effort if only two cameras can provide a substantial increase over one camera. Our work illustrates the benefit of such pilot studies to determine optimal survey design for camera trapping research, and highlights the substantial increase in data quality that results from a study design using transects with multiple cameras.

## Supporting information

S1 File Comparison between 24-hour and 1-week detection windows. (DOCX)

S2 File Results of phase 1 modeling to compare detection by method versus constant. (DOCX)

S3 File Model comparison between different possible pairs for methods with two cameras. (DOCX)
