# Supplementary material for: Assessing arrays of multiple trail cameras to detect North American mammals
Source: PLoS One. 2019 Jun 17;14(6):e0217543. doi: 10.1371/journal.pone.0217543 (PMC6576775; doi:10.1371/journal.pone.0217543)
Supplement: S1 File — (DOCX) [file pone.0217543.s001.docx]

**S1. Comparison between 24-hour and 1-week detection windows**

How camera trap data are delineated into “visits” will depend on study design and researcher objectives. With a relatively short time window to conduct our field work, we elected to place our survey stations for a minimum of two weeks and analyze each 24-hour window as a unique “visit”. From a qualitative assessment of our detection history data, the occasions with positive detections did not appear to be clumped in such a way that would suggest this 24-hour binning violated assumptions of temporal independence. However, we further assessed this by comparing the model fit and the results for Psi, theta and p_method from a weekly-binning approach instead, incorporating the same top model covariates.

Generally the trend in the effect that the method had on detection given availability was the same (S1 Fig), although absolute values of estimates varied (S1 Table). For coyote, the model was unable to converge on an estimate for Psi, and for weasel we see that the standard errors are noticeably larger than when running with the 24-hour detections.

**S1 Figure**

|  |  |
| --- | --- |
|  |  |
|  |  |

**S1 Table**

| **Species** | **Temporal bin** | $\hat{\text{Ѱ}}$**(se)** | $\hat{\boldsymbol{p}^{\boldsymbol{1}}}$**(se)** | $\hat{\boldsymbol{p}^{\boldsymbol{2}}}$**(se)** | $\hat{\boldsymbol{p}^{\boldsymbol{3}}}$**(se)** | $\hat{\boldsymbol{p}^{\boldsymbol{4}}}$**(se)** | $\hat{\boldsymbol{p}^{\boldsymbol{5}}}$**(se)** |
| --- | --- | --- | --- | --- | --- | --- | --- |
| *Canis latrans* | 24-hour | 0.67(0.17) | 0.13(0.07) | 0.51(0.09) | 0.65(0.08) | 0.34(0.09) | 0.62(0.09) |
|  | 1-week | ***Did not converge*** | 0.17(0.05) | 0.45(0.11) | 0.72(0.11) | 0.54(0.11) | 0.63(0.12 |
| *Pekania pennanti* | 24-hour | 0.73(0.07) | 0.60(0.16) | 0.79(0.11) | 0.83(0.09) | 0.71(0.14) | 0.73(0.13) |
|  | 1-week | 0.56(0.13) | 0.41(0.09) | 0.75(0.06) | 0.86(0.03) | 0.61(0.07) | 0.74(0.06) |
| *Martes americana* | 24-hour | 0.76(0.08) | 0.29(0.07) | 0.47(0.06) | 0.79(0.06) | 0.55(0.07) | 0.58(0.07) |
|  | 1-week | 0.79(0.09) | 0.41(0.08) | 0.79(0.05) | 0.89(0.03) | 0.72(0.05) | 0.75(0.05) |
| *Mustela erminea* | 24-hour | 0.51(0.12) | 0.24(0.05) | 0.47(0.06) | 0.60(0.06) | 0.52(0.06) | 0.77(0.05) |
|  | 1-week | 0.84(***0.25***) | 0.21(***0.15***) | 0.64(***0.36***) | 0.55(***0.33***) | 0.52(***0.31***) | 0.55(***0.33***) |
| *Lepus americanus* | 24-hour | 0.50(0.12) | 0.37(0.07) | 0.49(0.08) | 0.72(0.06) | 0.49(0.07) | 0.61(0.07) |
|  | 1-week | 0.51(0.14) | 0.29(0.16) | 0.59(0.13) | 0.75(0.09) | 0.44(0.16) | 0.50(0.15) |
| *Tamiasciurus hudsonicus* | 24-hour | 0.53(0.31) | 0.34(0.10) | 0.66(0.09) | 0.81(0.06) | 0.57(0.09) | 0.69(0.09) |
|  | 1-week | 0.57(0.32) | 0.32(0.13) | 0.78(0.10) | 0.75(0.12) | 0.59(0.15) | 0.63(0.14) |
| The different methods compared are: p^1^ is a single camera, p^2^ is two cameras spaced 100 m apart, p^3^ is two cameras spaced 150 m, p^4^ is three cameras spaced 100 m and p^5^ is three cameras spaced 150 m. | | | | | | | |
