# Supplementary material for: Assessing arrays of multiple trail cameras to detect North American mammals
Source: PLoS One. 2019 Jun 17;14(6):e0217543. doi: 10.1371/journal.pone.0217543 (PMC6576775; doi:10.1371/journal.pone.0217543)
Supplement: S2 File — (DOCX) [file pone.0217543.s002.docx]

**S2.** Results of phase 1 modeling to compare detection by method versus constant

The first step in our multi-scale occupancy model approach was to determine if our approach to pooling detection history data from different arrangements of cameras at the same survey site was valid. We did this by comparing a multi-scale model with no covariates, but with the detection parameter allowed to vary by each method p, to a model in which detection was held constant. In all cases the model including method ranked much higher (>∆AICc 8, with model weight always rounded to 1.00) as shown in S2 Table.

**S2 Table**

| **Species** | **Competing Models** | **AICc** | **∆AICc** | **AICw** |
| --- | --- | --- | --- | --- |
| *Canis latrans* | Ѱ(.), ϴ(.), p(Method) | 424.66 | 0 | 1.00 |
|  | Ѱ(.), ϴ(.), p(.) | 433.23 | 8.82 | 0.00 |
| *Pekania pennanti* | Ѱ(.), ϴ(.), p(Method) | 683.86 | 0 | 1.00 |
|  | Ѱ(.), ϴ(.), p(.) | 698.10 | 14.97 | 0.00 |
| *Martes americana* | Ѱ(.), ϴ(.), p(Method) | 1140.82 | 0 | 1.00 |
|  | Ѱ(.), ϴ(.), p(.) | 1179.25 | 38.43 | 0.00 |
| *Mustela erminea* | Ѱ(.), ϴ(.), p(Method) | 875.75 | 0 | 1.00 |
|  | Ѱ(.), ϴ(.), p(.) | 911.23 | 35.48 | 0.00 |
| *Lepus americanus* | Ѱ(.), ϴ(.), p(Method) | 1265.24 | 0 | 1.00 |
|  | Ѱ(.), ϴ(.), p(.) | 1324.72 | 59.08 | 0.00 |
| *Tamiasciurus hudsonicus* | Ѱ(.), ϴ(.), p(Method) | 575.87 | 0 | 1.00 |
|  | Ѱ(.), ϴ(.), p(.) | 588.35 | 12.48 | 0.00 |
