# Supplementary material for: Assessing arrays of multiple trail cameras to detect North American mammals
Source: PLoS One. 2019 Jun 17;14(6):e0217543. doi: 10.1371/journal.pone.0217543 (PMC6576775; doi:10.1371/journal.pone.0217543)
Supplement: S3 File — (DOCX) [file pone.0217543.s003.docx]

**S3. Model comparison between different possible pairs for methods with two cameras.**

The “2-short” method, with 100 m spacing, composed of the “Original” pair used in our analyses, cameras B and C in Fig. 2, and the “Alternative” pair, cameras A and B. The “2-Long” method with 150 m spacing is “Original” pair D and E, and “Alternative” pair B and C.

The Alternative pairs were used to create detection histories following the same methodology, and replaced the Original methods for 2-short and for 2-long. We ran the same multi-scale analysis (Nichols et al. 2008), and saw the same pattern for the support of “method” as a driving factor in the detection process (Table A in S3 File). We then compared the model output and observed the same trends in terms of 1, 2 and 3 cameras at the different spacing using both the Alternative and the Original pair (Table B in S3 File). Because the Original pair fully separates the detection histories used between method 1 (camera A) and 2-short, and between 2-short and 3-short, we carried out the rest of our analysis only using these pairs.

**Table A in S3 File**

| **Species** | **Pairs** | **Models** | **∆AICc** |
| --- | --- | --- | --- |
| *Canis latrans* | Original | Ѱ(.), ϴ(.), p(mOriginal)  Ѱ(.), ϴ(.), p(.) | 0  10.23 |
|  | Alternative | Ѱ(.), ϴ(.), p(mAlternative)  Ѱ(.), ϴ(.), p(.) | 0  8.82 |
| *Pekania penanti* | Original | Ѱ(.), ϴ(.), p(mOriginal)  Ѱ(.), ϴ(.), p(.) | 0  18.44 |
|  | Alternative | Ѱ(.), ϴ(.), p(mAlternative)  Ѱ(.), ϴ(.), p(.) | 0  10.43 |
| *Martes americana* | Original | Ѱ(.), ϴ(.), p(mOriginal)  Ѱ(.), ϴ(.), p(.) | 0  44.83 |
|  | Alternative | Ѱ(.), ϴ(.), p(mAlternative)  Ѱ(.), ϴ(.), p(.) | 0  37.78 |
| *Martes erminea* | Original | Ѱ(.), ϴ(.), p(mOriginal)  Ѱ(.), ϴ(.), p(.) | 0  38.43 |
|  | Alternative | Ѱ(.), ϴ(.), p(mAlternative)  Ѱ(.), ϴ(.), p(.) | 0  35.48 |
| *Lepus americanus* | Original | Ѱ(.), ϴ(.), p(mOriginal)  Ѱ(.), ϴ(.), p(.) | 0  26.97 |
|  | Alternative | Ѱ(.), ϴ(.), p(mAlternative)  Ѱ(.), ϴ(.), p(.) | 0  25.0 |
| *Tamiasciurus hudsonicus* | Original | Ѱ(.), ϴ(.), p(mOriginal)  Ѱ(.), ϴ(.), p(.) | 0  17.02 |
|  | Alternative | Ѱ(.), ϴ(.), p(mAlternative)  Ѱ(.), ϴ(.), p(.) | 0  12.48 |

**Table B in S3 File**

| **Species** | **Method** | **Original Pairs**  **Untransformed Beta Est.** | **Alternative Pairs**  **Untransformed Beta Est.** |
| --- | --- | --- | --- |
| *Canis latrans* | 1 camera | -1.632 | -1.599 |
|  | 2 short | 0.086 | 0.149 |
|  | 3 short | -0.307 | -0.255 |
|  | 2 long | -0.726 | 0.567 |
|  | 3 long | 0.627 | 0.716 |
| *Pekania pennanti* | 1 camera | -0.468 | -0.466 |
|  | 2 short | 0.537 | 0.389 |
|  | 3 short | 1.462 | 1.467 |
|  | 2 long | -0.125 | 0.314 |
|  | 3 long | 0.779 | 0.783 |
| *Martes americana* | 1 camera | -0.578 | -0.575 |
|  | 2 short | 0.122 | 0.442 |
|  | 3 short | 0.776 | 0.782 |
|  | 2 long | 0.438 | 0.651 |
|  | 3 long | 1.582 | 1.593 |
| *Martes erminea* | 1 camera | -1.185 | -1.179 |
|  | 2 short | 0.001 | -0.183 |
|  | 3 short | 0.402 | 0.413 |
|  | 2 long | -0.191 | 0.362 |
|  | 3 long | 1.077 | 1.094 |
| *Lepus americanus* | 1 camera | -1.001 | -1.018 |
|  | 2 short | -0.520 | -0.464 |
|  | 3 short | 0.347 | 0.318 |
|  | 2 long | -0.082 | -0.648 |
|  | 3 long | 0.384 | 0.342 |
| *Tamiasciurus hudsonicus* | 1 camera | -1.225 | -1.221 |
|  | 2 short | 0.0389 | -0.132 |
|  | 3 short | 0.586 | 0.596 |
|  | 2 long | -0.318 | 0.135 |
|  | 3 long | 0.785 | 0.797 |
